# Possible use of organic compounds on shelf life and quality properties of peeled pomegranate

**DOI:** 10.1002/fsn3.1351

**Published:** 2019-12-17

**Authors:** Hamed Kaveh, Safieh Vatandoost

**Affiliations:** ^1^ Department of Plant Production University of Torbat Heydarieh Iran; ^2^ Saffron Institute University of Torbat Heydarieh Iran

**Keywords:** “Ardestani”, *Aloe vera*, aril quality, customer acceptance, saffron extract

## Abstract

Pomegranate cultivar (“Ardestani”) peeled and packed in polyethylene containers and treated with different natural products. Two concentrations of *Aloe vera* gel (10 and 15%), two different levels of saffron petal extracts (10 and 20% V/V) and two concentrations of saffron style extract (0.1 and 1% V/V) and control in one storage condition (7°C and 85% RH) were the treatments that applied by a full factorial randomized method. We examined natural substances for their possible application in extending the shelf life of fresh‐cut horticultural products to find a new approach for packaging and exporting pomegranates. About 13.8% mass loss in the 12th day of storage occurred because of higher enzymatic activity and lower membrane resistance. Our results show that all treatments significantly reduced mass loss, and *Aloe vera* gel treatments combined with saffron petal extract were the best. Although all treatments decreased ion leakage, *Aloe vera* gel and saffron petal extract reduced it significantly. Ion leakage incidence of arils at day 12 was lower in *Aloe vera* gel and saffron petal extract treatment compare to control. Application of both saffron extracts on arils reduced decay incidence and chilling injury from 86.67% to 6.67% and 60% to 26.67%, respectively. Total acidity, soluble solids content, total phenol content, anthocyanin content, and antioxidant capacity of arils changed differently in different treatments, and saffron petal extract significantly was the best one and increased anthocyanin content, total phenol content, and antioxidant capacity in arils. The microbial contamination increased in more extended storage, although both saffron extracts were successfully suppressed mold and bacteria growth below acceptable limits in 14 days at 7°C.

## Graphical abstract

Application of Agar Gel and saffron extracts reduced weight loss of fresh‐cut pomegranate arils. Both saffron extracts suppressed fungi and bacteria development on processed pomegranate fruits, successfully, so they can be used as natural preservatives. Finally and after 14 days of storage at 7°C and 85 % RH, arils with combined application of SPE and SSE had better consumer acceptance.

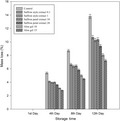

## 1 INTRODUCTION

Pomegranate (*Punica granatum* L), which is probably native to Iran or northern Turkey (Levin, 1994; Ward, 2003), is one of the most popular fruits around the world. It contains a high level of antioxidants that may affect the desired blood parameters and preventing various coronary arteries and some types of cancers (Lansky, Shubert, & Neeman, 2000; Noitsakis, Chouzouri, Papa, & Patakas, 2016). The problems of peeling and extracting pomegranate arils have limited its use. For this reason, the production and availability of fresh pomegranate arils may increase the consumption of fresh pomegranate (Gil, Martınez, & Artés, 1996; Nunes, Graça, Yıldırım, Sahin, & Erkan, 2009). The storage life of freshly cut arils is shorter than the whole fruit, and further studies are needed to extend it (López‐Rubira, Conesa, Allende, Artés, & Technology, 2005). The main problems affecting the overall quality of fresh‐cut pomegranates are microbial growth and activity of the browning enzyme, which is due to the oxidation of phenolic compounds by polyphenol oxidase (Gil et al., 1996).

Fruits and vegetables are metabolically active and subjected to senescence changes that need to be controlled to maintain long‐term quality and shelf life (Mahajan, Caleb, Singh, Watkins, & Geyer, 2014). The use of postharvest technologies has proven effects on mortality reductions of fresh produce in different parts of the world and can be a strategic procedure for reducing poverty, hunger, and malnutrition (Affognon, Mutungi, Sanginga, & Borgemeister, 2015). The choice of postharvest technology depends on the type of product, climatic conditions on production and transportation, pricing, and usability (Kitinoja & Barrett, 2015). The goal of postharvest technology is to reduce the rate of physiological reactions and senescence, and thus minimizing product decomposition. Some postharvest techniques include the use of chemical and physical methods that are effective in reducing microbial contamination (Tripathi, Sharma, Sharma, & Alam, 2013). Adding chemicals to keep food products safe and sound is usually based on preventing microbial growth or killing and destroying harmful microorganisms. Regarding the general concerns about the effects of chemical preservatives to human health, consumers tend to buy and use products that have no preservatives or natural preservatives.

Many studies have been carried out on natural preservatives in recent years. Herbal extracts, essential oils, and their constituent parts have known for antibacterial and antioxidant effects (Canillac & Mourey, 2001; Kaveh, 2017; Parray, Kamili, Hamid, Reshi, & Qadri, 2015). Phytochemical content of saffron petals and stigma (Montoro, Tuberoso, Maldini, Cabras, & Pizza, 2008; Nørbæk, Brandt, Nielsen, Ørgaard, & Jacobsen, 2002) is flavonoids, anthocyanins, alkaloids, carbohydrate glycosides, tannins, terpenes, steroids, and saponins which was useful in extending shelf life of fresh‐cut fruits like watermelon (Kaveh, 2017).

The most used part of saffron (*Crocus sativus* L.), which is widely cultivated in Iran as the most expensive spice of the world, is the *stigma*. Due to its numerous applications in the food and pharmaceutical industries, only *stigma* looks fit for manufacturing purposes and almost 90% of the harvested parts of the flower usually abandoned as waste without any proper usage, although phytochemical components and potential application of other saffron floral parts in the health and food industries considered by the scientific community (Serrano‐Díaz et al., 2012). So far, different amounts of carbohydrates such as glucose, fructose, sucrose, maltose, inositol, sorbitol and mannitol (Serrano‐Díaz, Sánchez, Martínez‐Tomé, Winterhalter, & Alonso, 2013), proteins, lipids, fiber, mineral elements (phosphorus, magnesium, calcium, iron, potassium) (Serrano‐Díaz et al., 2013), volatile and aroma (boto‐lactone and 2,3‐butanediol) (Argento et al., 2009; Zheng, Li, Ma, Han, & Qin, 2011), carotenoid derivatives (crocetin, crocin, and lutein di‐esters) (Goupy, Vian, Chemat, & Caris‐Veyrat, 2013; Tuberoso, Rosa, Montoro, Fenu, & Pizza, 2016), picrocrocin, croco satin and phenolic compounds like benzoic acid, hydroxy‐cinnamic acid, anthocyanins, and flavonoids (Serrano‐Diaz, Sanchez, Martinez‐Tome, Winterhalter, & Alonso, 2014) are reported in saffron petals. The biological activity of saffron petal extract has been studied and proved its antityrosinase (Yildiztekin et al., 2016), antioxidant (Sánchez‐Vioque et al., 2012; Serrano‐Díaz et al., 2012; Termentzi & Kokkalou, 2008; Yildiztekin et al., 2016) antifungal (Zheng et al., 2011), and antimicrobial (Kaveh, 2017) effects. Saffron stigma, which is famous for its color, taste, and odor, has special antibacterial effects, which is mainly due to *safranal*.

## 2 MATERIALS AND METHODS

### 2.1 Preparation of plant materials

Mature, uniform (similar in shape and size) "*Ardestani*" pomegranate fruits were harvested from commercial Orchard, Mahvelat, and Khorasan‐Razavi province in autumn 2018 and transported to the Laboratory. Fruits carefully examined in terms of being free of pests and diseases, skin lesions, and signs of sunburn. Fresh, unharmed fruits peeled and seeded.

### 2.2 Treatments application

Pomegranate arils divided into four groups for treatment application. Treatments included different levels of *Aloe vera* gel (AG) (0, 10 and 15%), saffron petal extracts (SPE) (0, 10 and 20% V/V), and saffron style extract (*SSE*) (0, 0.1 and 1% V/V). Arils of each group then weighted (100 gr) and packed in low‐density polyethylene bags. All packages stored in 7 degrees celsius and 90% relative humidity for 12 days.

This experiment conducted as factorial in a completely randomized design with three replications. Each replication contains ten packages (each one with 100 gr pomegranate aril) as observation, and the data for each replication are mean of them.

### 2.3 Sampling, measurements, and observations

In this study, arils mass loss, ion leakage, chilling injury and decay percentage, soluble solids content, titratable acidity, anthocyanin content, total phenol, antioxidant capacity, and microbial contamination of minimally processed pomegranate arils measured.

### 2.4 Mass loss

Weighting each package during storage, the *mass loss* was calculated and expressed in percentage (Eq. 1).

(1) $$\text{ML}=\left[\left(W_{i1}-W_{\mathrm{it}}\right)/W_{i1}\right]*100$$

where W_i1_ stands for package weight at the beginning of the experiment, and W_it_ stands for package weight during storage period on sampling.

### 2.5 Ion leakage (IL)

*Ion leakage* (IL) was calculated with four grams sample for each packet. It was then stored in a becher containing 20 ml of water for 24 hr, and then, the initial electrical conductivity (EC1) was read by Ec meter. The samples then placed in Ben‐Marie at 100°C for one hour and, after cooling down to room temp., the secondary electrical conductivity (EC2) was measured. Finally, ion leakage was determined using equation No.2 (Barranco, Ruiz, & Gómez‐del Campo, 2005).

(2) $$\text{Ion Leakage Percentage}=\left[\left(\text{EC2}-\text{EC1}\right)/\text{EC2}\right]*100$$

### 2.6 Decadence/Chilling injury

*Decadence/chilling injury* (DI/CI) percentage of pomegranate arils was measured by observing and counting the number of packages with decayed/injured arils incidence and calculating relative to the total number of packets according to the following equation (Karabulut, Gabler, Mansour, & Smilanick, 2004).

(3) $$\text{Decay}/\text{CI Percentage}=\left[{\text{NP}}_{i}/{\text{NP}}_{t}\right]*100$$

NP_i_: number of packets with decay/CI incidence at sampling time (in each specific treatment), and NP_t_: number of total packets in each treatment. The data are reported cumulatively between observations. If a treatment has 10 percent of decay in first observation and 10 percent in the second one, 20 percent of decadence in second observation was reported. Also, it should be cleared that one package may show both decay or chilling injury incident and counted in both terms in each time.

### 2.7 Titrable acidity

Pomegranate arils fresh juice was used to measure *titrable acidity* (TA) according to the described method by Ayala‐Zavala, Wang, Wang, and González‐Aguilar (2005); Belay, Caleb, Mahajan, and Opara (2018). The TA content of samples was measured potentiometrically by titration with 0.1 mol/LNaOH, to an end‐point of pH 8.2. The TA value was expressed as milligrams per liter of citric acid based on fresh weight.

### 2.8 Anthocyanin content

*Anthocyanin content* (AC) of pomegranate juice measured using the pH differential method according to Belay et al., 2018; Lako et al. (2007) method. Nine ml potassium chloride buffer for pH 1.0 (0.025 M) and sodium acetate buffer for pH 4.5 (0.4 M) was used separately to dilute each sample (1 ml of fresh juice). After 10 min, absorbance was observed at 510 and 700 nm in pH 1.0 and 4.5 buffers. Results were calculated with Equation No.4 and expressed as cyanidin‐3‐glucoside equivalents.^1^

(4) $$\text{Total anthocyanin}\;\left(\mu gL^{-1}\right)\;=\;\left[\left(A_{\mathrm{abs}}*449.2*9*1000\right)/26900\right]*L$$

where A_abs_ stands for A520–A700, 449.2 g/mol is the molecular weight of cyanidin‐3‐glucoside, 9 is the dilution factor, 26.900 is molar extinction coefficient, and L represents path length in centimeters.

### 2.9 Soluble solids content

*Soluble solid*s *content* (SSC) of pomegranate juice was measured using a hand refractometer (Atago™ MASTER‐53M) and expressed as percent.

### 2.10 Total phenol content and antioxidant capacity

*Total phenol content* (TPC) and *antioxidant capacity* (ACP) were measured according to the method described by Du, Li, Ma, and Liang (2009). Five grams of pomegranate arils was extracted and smashed in liquid nitrogen and then 20 ml ethanol: Acetone (7/3 v/ v) solution was added to the sample. After homogenization, it was placed at room temperature for one hour and then filtered with Watten's No. 4 filter paper. Total phenol and total antioxidant capacity were determined from the extracted solution.

**Figure 1:**
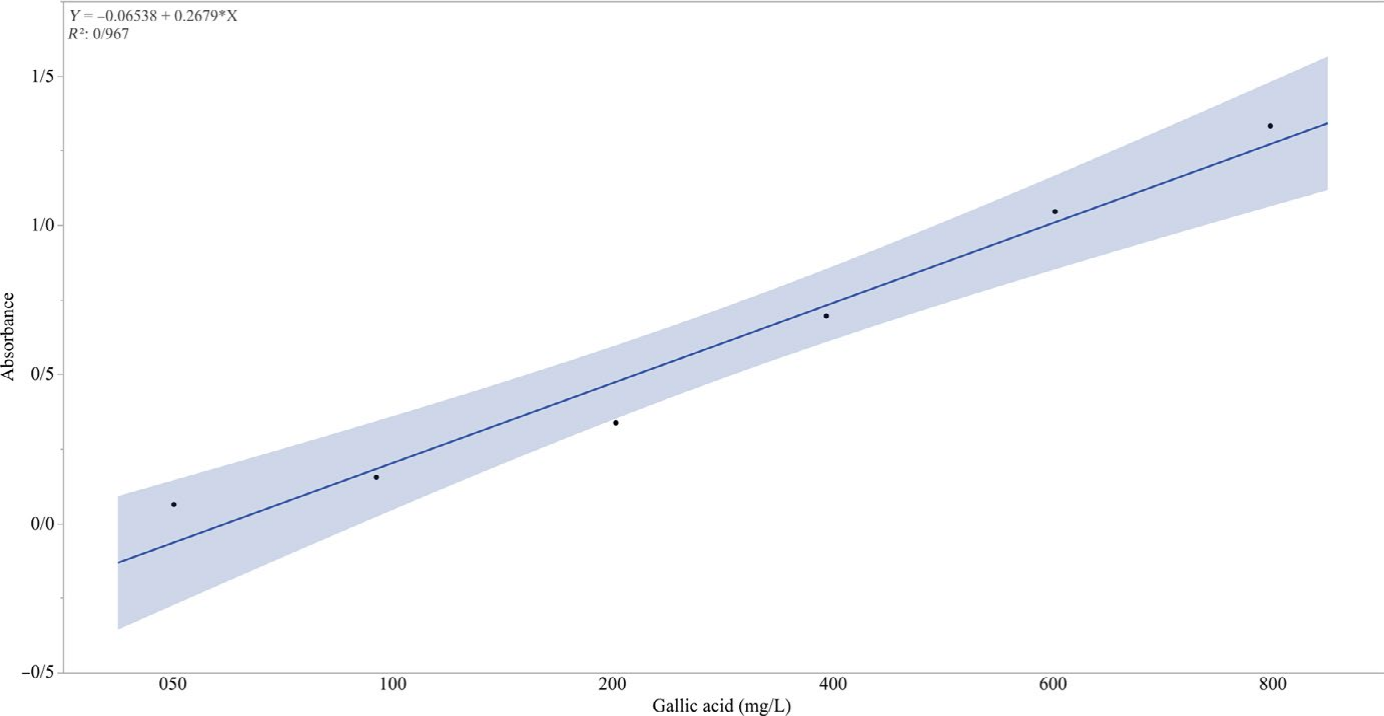
Gallic acid standard curve and equation

Total phenol content was measured according to Folin–Ciocalteu method using a spectrophotometer (Du et al., 2009). A 5 ml of sample (200 μl of the aril extract plus distilled water) was added to 500 μl of Folin (1:1 with water); then, 1,500 μl of sodium carbonate (20 g/ 100 ml) was added after one minute. After two hours of storage at room temperature in no light condition, the absorbance of the extract was measured at 765 nm. Pure gallic acid was used to obtain the standard curve. A 100 μl of the prepared solution of gallic acid at concentrations of 0–1000 with 0.5 ml of Folin 50% and 1.5 ml of sodium carbonate 20% was mixed and kept in darkness for 2 hr; then, absorbance at 765 nm was read, and then, the standard curve was plotted (Figure 1). Before measuring the samples, the device calibrated with a blank sample containing 100 μl of extraction solvent, 9.9 ml of water, 0.5 ml of Folin (50%), and 1.5 ml of sodium carbonate (20%). This experiment was carried out on samples in three replicates. Finally, the total phenol content was calculated from the absorbance of the sample and standard samples per milligram of gallic acid in five grams of fresh tissue.

Extracts antioxidant capacity was measured by the inhibitory capacity of 2,2‐diphenyl‐1‐picrylhydrazyl (DPPH), according to Dow et al. (2009). So, 200 μl of aril extract was poured into a small Falcon tube and added to 1,800 μl of DPPH (6.25*10^−5^). The solution was quickly stirred up and then stored at room temperature, no light, for 30 min. The sample and standard solutions absorbance were read by spectrophotometer at 515 nm. Finally, the antioxidant capacity of the extracts was calculated as the DPPH inhibitory concentration according to the following equation (Eq. No.5).

(5) $$\%{\text{DPPH}}_{\text{sc}}=\left(A_{\text{CONT}}-A_{\text{SAMP}}\right)/A_{\text{CONT}}*100$$

where % DPPH_sc_ is the inhibitory percentage of DPPH, A_CONT_ is absorbance of DPPH at 515 nm, and A_SAMP_ is sample absorbance at 515 nm.

### 2.11 Microbial quality

*Microbial quality* of pomegranate arils was studied according to methods described by Belay et al., 2018. 10 g of pomegranate arils was mixed with 90 ml peptone buffered the water and homogenized for 2 min with a laboratory blender. Plate count agar (PCA) was used for the aerobic bacterial count, while rose bengal chloramphenicol agar (RBCA) was used to count mold. PCA plates incubated at 30°C for three days and RBCA plates at 25°C for five days. After incubation, colonies were counted on each plate, and the results were expressed as log colony‐forming unit per weight (log CFU/ml) (Belay et al., 2018).

### 2.12 Data collection

Data collection and measurements were done at the 1st, 4th, 8th, and 12th day of the experiment. Then, data were subjected to analysis of variance. Tukey HSD multiple range test at 95% confident interval with SAS‐JMP (ver. 14) was the tool for evaluation of the difference between mean values significance.

## 3 RESULTS

### 3.1 Mass loss

**Figure 2:**
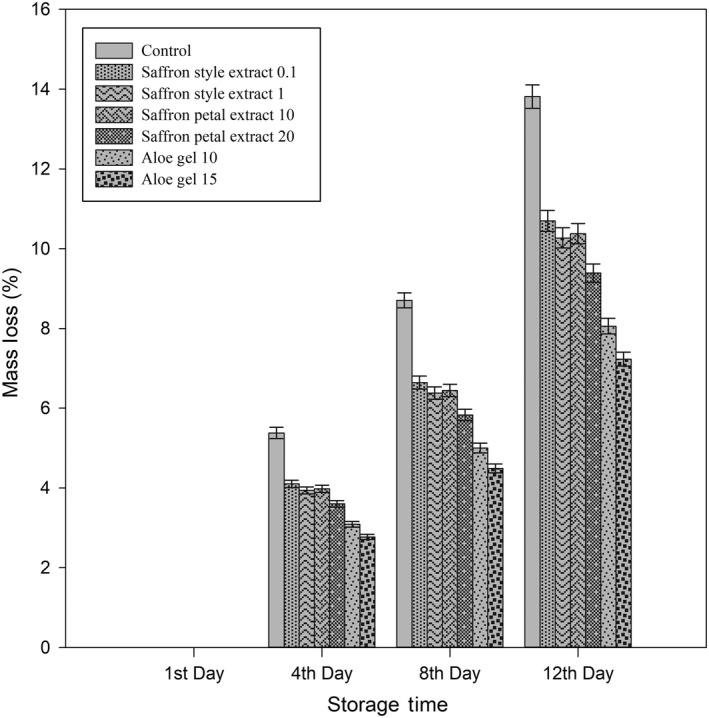
Effects of different treatments on mass loss in pomegranate arils during storage. Each error bar is constructed using a 95% confidence interval of the mean according to Tukey HSD multiple range test

**Figure 3:**
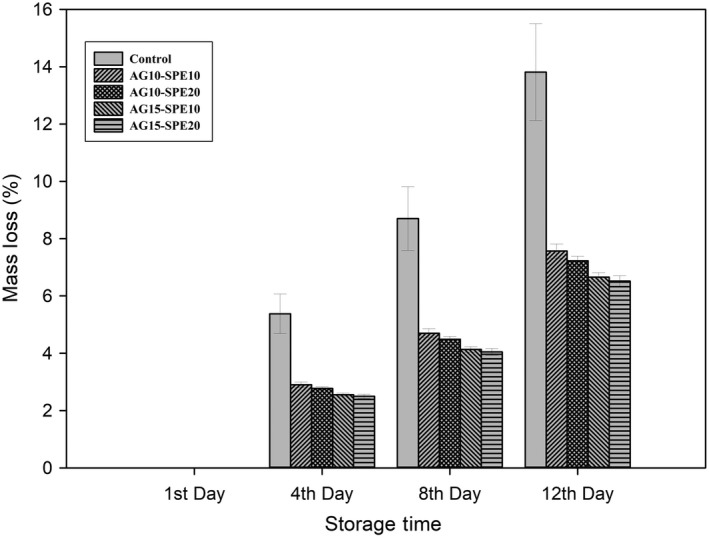
Effects of saffron petal extract (10 and 20%) and *Aloe vera* gel (10 and 15%) interaction on mass loss in pomegranate arils during storage. Each error bar is constructed using a 95% confidence interval of the mean according to Tukey HSD multiple range test

**Figure 4:**
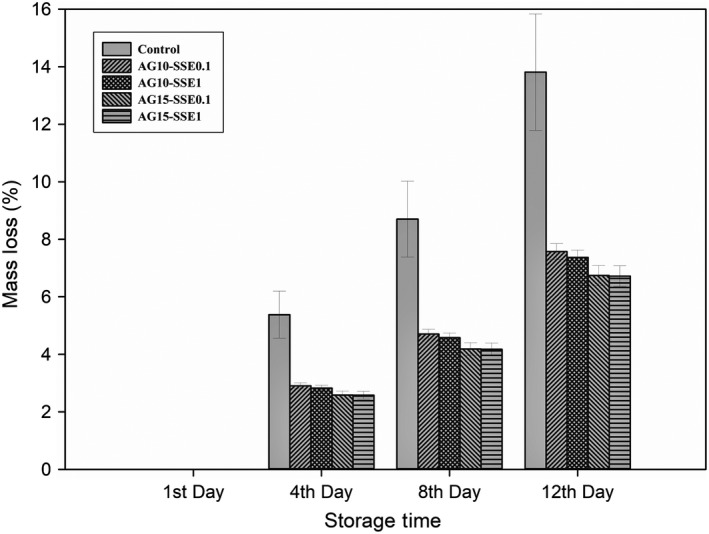
Effects of saffron style extract (0.1 and 1%) and *Aloe vera* gel (10 and 15%) interaction on mass loss in pomegranate arils during storage. Each error bar is constructed using a 95% confidence interval of the mean according to Tukey HSD multiple range test

Schematic weight loss of treated arils kept at 7°C was showed in Figure 2. By the rise in the concentration of treatments, reduction in mass loss observed at each sampling time. During each observation, AG 15% had the lowest mass loss, and AG 10% and SPE 20% were in next places (*p* < .05). Although all treatments were reduced mass loss significantly (*p* < .05) in comparison with control, differences between *SSE* 0.1%, *SSE* 1%, and SPE 10% were not significant. Application of 0.1 and 1% *SSE* reduced weight loss of control treatment at the 12th day of storage from 13.8% to 10.69% and 10.27%, respectively (*p* < .05). Different studies reported that more extended storage periods of pomegranate arils cause higher weight loss due to more enzymatic activity and lower cell membrane resistance against water loss *(*Atilgan et al., 2014; Belay et al., 2018). Combined application of Aloe gel with saffron petal and style extracts had the same trend (Figures 3 and 4), while SPE was better in mass loss reduction. Aloe Gel controls micro atmospheric exchanges of O_2_ and Co_2_ in treated arils, while the antioxidant activity of saffron extracts may reduce oxidation ratio and enzymatic activity. Results suggest that Aloe Gel's treatment was the most effective weight loss prevention treatment (Figure 2), which is better to be combined with SPE 20% in the application (Figure 3).

Essential oils application on postharvest of fresh‐cut apple was showed that as their treatment cannot provide a lipid form, multilayer coating on fresh‐cut fruits, they could not be able to reduce mass loss, while chitosan and pectin edible coatings were effectual in papaya and pineapple (Sarengaowa, Hu, Jiang, Xiu, & Feng, 2018). Different reports suggest that coating pomegranate fruit or arils with *Aloe vera*, starch with or without oil combination (glycerol plus *Oleum nigella*), and lecithin provides a louver to water exchange between product and atmosphere, which reduces the mass loss (Opara, Atukuri, & Fawole, 2015).

### 3.2 Ion leakage

**Figure 5:**
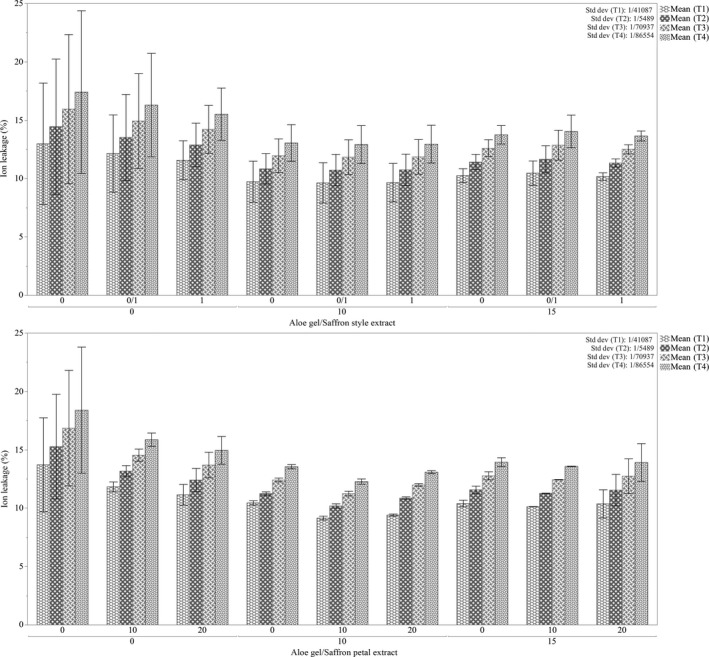
Effects of different treatments on electrolyte leakage in pomegranate arils on each storage period (Up: effects of *Aloe Vera* Gel and saffron style extract interaction on ion leakage, Down: effects of *Aloe vera* Gel and saffron petal extract interaction on Ion leakage. T1: day 1, T2: day 4, T3: day 8, T4: day 12).Each error bar is constructed using a 95% confidence interval of the mean according to Tukey HSD multiple range test

An increased rate of electrolyte leakage has been used as an indicator of physical damage to cell membranes during low‐temperature storage of horticultural produces. Electrolyte leakage measures the integrity of plant cells and tissues, and an increase in EL indicates deterioration in cellular membrane systems. As it is showed in Figure 5, AG concentration significantly decreased IL in compare to control, while there were no significant differences between 10% and 15% application of Aloe Gel (*p* ≤ .05). *SSE* was not useful in ion leakage reduction, while saffron petal extract 20% significantly reduced ion leakage.

In all treatments, it is clear that the storage of arils for longer times will cause higher electrolyte leakage (Figure 5).

In papaya, ethylene suppressors could lower electrolyte leakage of fresh‐cut fruits by lowering the respiration rate (Muharrem, Donald, Jiwon, & Jerry, 2006). The alginate‐based coating containing 0.05% ε‐PL significantly reduced electrolyte leakage of kiwi fruits (Li et al., 2017). Modified atmosphere packaging was also recognized as a useful tool in IL reduction of different fresh‐cut produces during cold storage. It was also beneficial to use MAP in the storage of intact pomegranate fruits for long‐term storage (Valdenegro et al., 2018).

### 3.3 Decadence/Chilling injury

Since pomegranate is a subtropical fruit, chilling injury and decay of arils may be a significant problem during produce marketing, especially when they were kept in relatively low temperatures.

In different studies, researchers try to lower CI during fruit storage by means of *Putrescine* (Barman, Asrey, & Pal, 2011), *MAP* (Artés, Villaescusa, & Tudela, 2000; López‐Rubira et al., 2005), *salicylic acid (SA)* (Sayyari, Babalar, Kalantari, Serrano, & Valero, 2009), *arginine* (Babalar, Pirzad, Sarcheshmeh, Talaei, & Lessani, 2018), *heat treatment* (Yanclo, 2018), *gamma irradiation* (Ashtari, Khademi, Soufbaf, Afsharmanesh, & Askari Sarcheshmeh, 2019), and nitric oxide (Ranjbari, Moradinezhad, & Khayyat, 2018).

Using a more environment‐friendly technique, which has no remaining residue, may be more favorable. Here, the results of this study reveal that 10% of Aloe Gel treatment significantly reduce CI (Table 1). In the first and fourth days of the experiment, no CI or decay incident was observed. At day 8th, both saffron petal and style extracts reduced CI and decay occurrence, 43.34% to 13.34% and 40% to 20%, respectively (Table 1). This reduction may relate to the antioxidant and antimicrobial effects of saffron extracts.

**Table 1:** Effect of different treatments on aril decay and chilling injury

Storage time (DAYS)	Aloe Gel (%)	Saffron petal Extract	Mean decay occurrence (%)			Mean chilling injury (%)		
			*SSE*(0)	*SSE*(0.1)	*SSE*(1)	*SSE*(0)	*SSE*(0.1)	*SSE*(1)
8	0	0	43.34	20	13.34	40	30	20
		10	20	10	10	30	30	20
		20	13.34	3.34	0	20	20	6.67
	10	0	33.34	20	10	20	20	6.67
		10	20	20	10	20	6.67	6.67
		20	20	10	3.34	6.67	6.67	6.67
	15	0	40	26.67	20	6.67	6.67	6.67
		10	30	20	10	6.67	6.67	6.67
		20	20	13.34	3.34	6.67	6.67	6.67
12	0	0	86.67	30	23.34	60	50	40
		10	30	20	20	50	50	40
		20	23.34	13.34	6.67	40	40	26.67
	10	0	50	30	20	40	40	26.67
		10	30	30	20	40	26.67	26.67
		20	30	20	16/67	26.67	26.67	26.67
	15	0	50	36.67	30	26/67	26/67	26.67
		10	36.67	30	20	26/67	26/67	26.67
		20	26.67	20	13.33	26/67	26/67	26.67

Note

Means are significantly different at *p* ≤ 5% with a difference more than 4/41236 according to Tukey HSD multiple range test.

Abbreviation: SSE, saffron style extract.

### 3.4 Biochemical composition

Total acidity, SSC, total phenol content, anthocyanin content, and antioxidant capacity of treated pomegranate arils are demonstrated in Table 2 and Table 3. TA (mg.L^−1^) decreased through storage time significantly, but SSC (%) had not been affected by storage duration significantly. Similarly, Penastevez *et al*. reported no significant changes in SSC in arils during storage, while TA changed in different treatments in 5°C storage for 14 days (Peña‐Estévez et al., 2015). Higher Aloe Gel percentage reduced TA and increased SSC in each storage period. The reduction was significant between control and AG 10%, nor for the AG 10 and 15%. Saffron petal and style extracts increased TA significantly in comparison with control (Table 2).

**Table 2:** Effect of storage duration and different concentrations of Aloe Gel, saffron petal, and style extracts on TA (mg/l) and SSC (%) of pomegranate arils

Treatments			Total acids (mg/l)				Soluble solids content (%)			
AG	SPE	*SSE*	T1	T2	T3	T4	T1	T2	T3	T4
0	0	0	626.60	642.05	603.04	578.44	18.07	18.50	18.32	18.37
		0.1	638.49	653.52	615.02	585.15	17.83	18.26	18.08	18.13
		1	639.70	654.58	615.94	585.95	17.98	18.41	18.23	18.27
	10	0	638.10	650.54	611.16	580.47	19.37	19.84	19.64	19.69
		0.1	650.82	663.54	623.38	592.09	19.74	20.21	20.02	20.07
		1	655.64	668.20	627.65	596.04	20.04	20.52	20.32	20.37
	20	0	652.49	662.56	621.36	589.11	21.40	21.92	21.71	21.76
		0.1	664.02	675.83	634.44	602.13	20.85	21.35	21.14	21.19
		1	670.16	681.90	640.07	607.39	21.15	21.66	21.45	21.50
10	0	0	621.64	635.10	597.20	567.74	18.07	18.50	18.32	18.37
		0.1	638.89	653.95	615.43	585.54	17.83	18.26	18.08	18.13
		1	640.10	655.01	616.35	586.34	17.98	18.41	18.23	18.27
	10	0	638.49	650.96	611.56	580.86	19.37	19.84	19.64	19.69
		0.1	651.22	663.97	623.80	592.49	19.74	20.21	20.02	20.07
		1	656.04	668.63	628.07	596.44	20.04	20.52	20.32	20.37
	20	0	652.88	662.99	621.77	589.51	21.40	21.92	21.71	21.76
		0.1	664.43	676.26	634.86	602.53	20.85	21.35	21.14	21.19
		1	670.57	682.34	640.49	607.80	21.15	21.66	21.45	21.50
15	0	0	620.55	633.93	596.08	566.64	18.07	18.50	18.32	18.37
		0.1	637.75	652.73	614.26	584.41	17.83	18.26	18.08	18.13
		1	638.97	653.79	615.18	585.21	17.98	18.41	18.23	18.27
	10	0	637.37	649.77	610.41	579.74	19.37	19.84	19.65	19.69
		0.1	650.08	662.75	622.62	591.35	19.74	20.22	20.02	20.07
		1	654.89	667.40	626.89	595.30	20.04	20.52	20.32	20.37
	20	0	651.75	661.77	620.60	588.38	21.40	21.92	21.71	21.76
		0.1	663.27	675.02	633.67	601.38	20.85	21.35	21.14	21.19
		1	669.40	681.08	639.28	606.64	21.15	21.66	21.45	21.50

Note

Means of each studied trait are significantly different at *p* ≤ 5% with a difference more than 4/41236 according to Tukey HSD multiple range test.

Abbreviations: SPE, saffron petal extracts; SSE, saffron style extract; SSC, Soluble solids content.

**Table 3:** Effect of storage duration and different concentrations of *Aloe* Gel, Saffron Petal, and Style extracts on Anthocyanin content (ug/l) and total phenol content and antioxidant capacity of pomegranate arils

Treatments			Anthocyanin content (ug.L^−1^)				Total phenol content (mg.L^−1^)				Antioxidant capacity (%)			
AG	SPE	*SSE*	T1	T2	T3	T4	T1	T2	T3	T4	T1	T2	T3	T4
0	0	0	216.81	212.77	167.77	116.74	1,663.00	2,676.45	2,847.44	3,024.02	82.60	76.81	57.38	39.92
		0.1	213.96	209.98	165.57	115.21	1,701.50	2,730.87	2,909.53	3,061.96	81.52	75.80	56.63	39.40
		1	215.71	211.70	166.93	116.15	1,703.40	2,733.91	2,912.77	3,065.37	82.19	76.42	57.09	39.72
	10	0	232.43	228.10	179.86	125.15	1,681.86	2,699.35	2,875.94	3,026.61	88.56	82.35	61.51	42.80
		0.1	236.87	232.46	183.30	127.54	1,715.59	2,753.48	2,933.62	3,087.31	90.25	83.92	62.69	43.62
		1	240.47	235.99	186.09	129.48	1,726.44	2,770.90	2,952.19	3,106.85	91.62	85.19	63.64	44.28
	20	0	256.82	252.04	198.73	138.28	1,700.64	2,729.49	2,908.06	3,060.41	97.85	90.99	67.97	47.29
		0.1	250.17	245.51	193.59	134.70	1,741.90	2,795.72	2,978.62	3,134.67	95.31	88.63	66.21	46.07
		1	253.77	249.04	196.37	136.64	1,756.72	2,819.49	3,003.95	3,161.33	96.68	89.90	67.16	46.73
10	0	0	216.81	212.78	167.78	116.74	1,648.11	2,645.18	2,818.24	2,965.89	82.61	76.81	57.38	39.93
		0.1	213.97	209.99	165.58	115.21	1,702.69	2,732.78	2,911.57	3,064.10	81.52	75.81	56.63	39.40
		1	215.72	211.70	166.93	116.15	1,704.59	2,735.82	2,914.81	3,067.51	82.19	76.43	57.09	39.72
	10	0	232.43	228.11	179.87	125.15	1,683.03	2,701.23	2,877.95	3,028.73	88.56	82.35	61.51	42.80
		0.1	236.88	232.47	183.31	127.55	1,716.79	2,755.40	2,935.67	3,089.47	90.25	83.92	62.69	43.62
		1	240.48	236.00	186.09	129.48	1,727.65	2,772.84	2,954.25	3,109.02	91.62	85.20	63.64	44.28
	20	0	256.82	252.04	198.74	138.28	1,701.83	2,731.40	2,910.09	3,062.55	97.85	90.99	67.97	47.29
		0.1	250.18	245.52	193.60	134.70	1,743.12	2,797.67	2,980.70	3,136.86	95.32	88.63	66.21	46.07
		1	253.77	249.05	196.38	136.64	1,757.94	2,821.46	3,006.05	3,163.54	96.69	89.91	67.16	46.73
15	0	0	216.82	212.78	167.78	116.74	1,644.82	2,639.89	2,812.60	2,959.95	82.61	76.82	57.38	39.93
		0.1	213.97	209.99	165.58	115.21	1,699.28	2,727.31	2,905.74	3,057.97	81.52	75.81	56.63	39.40
		1	215.72	211.71	166.94	116.15	1,701.18	2,730.35	2,908.98	3,061.38	82.19	76.43	57.09	39.72
	10	0	232.44	228.12	179.87	125.16	1,679.67	2,695.83	2,872.20	3,022.67	88.56	82.35	61.52	42.80
		0.1	236.89	232.48	183.31	127.55	1,713.35	2,749.89	2,929.80	3,083.29	90.25	83.92	62.69	43.62
		1	240.48	236.01	186.10	129.49	1,724.19	2,767.30	2,948.34	3,102.80	91.62	85.20	63.64	44.28
	20	0	256.83	252.05	198.75	138.29	1,698.42	2,725.93	2,904.27	3,056.42	97.85	90.99	67.97	47.29
		0.1	250.18	245.53	193.60	134.71	1,739.63	2,792.07	2,974.74	3,130.58	95.32	88.63	66.21	46.07
		1	253.78	249.06	196.38	136.64	1,754.43	2,815.82	3,000.04	3,157.21	96.69	89.91	67.16	46.73

Note

Means of each studied trait are significantly different at *p* ≤ 5% with a difference more than 4/41236 according to Tukey HSD multiple range test.

Abbreviations: SPE, saffron petal extracts; SSE, saffron style extract.

Anthocyanin content and antioxidant capacity of pomegranate arils decreased, and total phenol content increased significantly in longer storage. *Aloe vera* Gel did not affect AC (ug.L^−1^) and ACP (%) significantly in each storage period, while TPC (mg.L^−1^) decreased significantly in the higher percentage of Aloe Gel treatments.

Applying different concentrations of saffron petal extract, which contains anthocyanin, on pomegranate arils, increased AC, TPC, and ACP significantly (*p* ≤ 5%) (Table 3), but saffron style extract was not effective in changing those chemical characteristics of arils significantly (*p* ≤ 5%). In the application of saffron petal extracts on fresh‐cut watermelon, similar findings in increasing AC were reported (Kaveh, 2017).

### 3.5 Microbial contamination analysis

**Figure 6:**
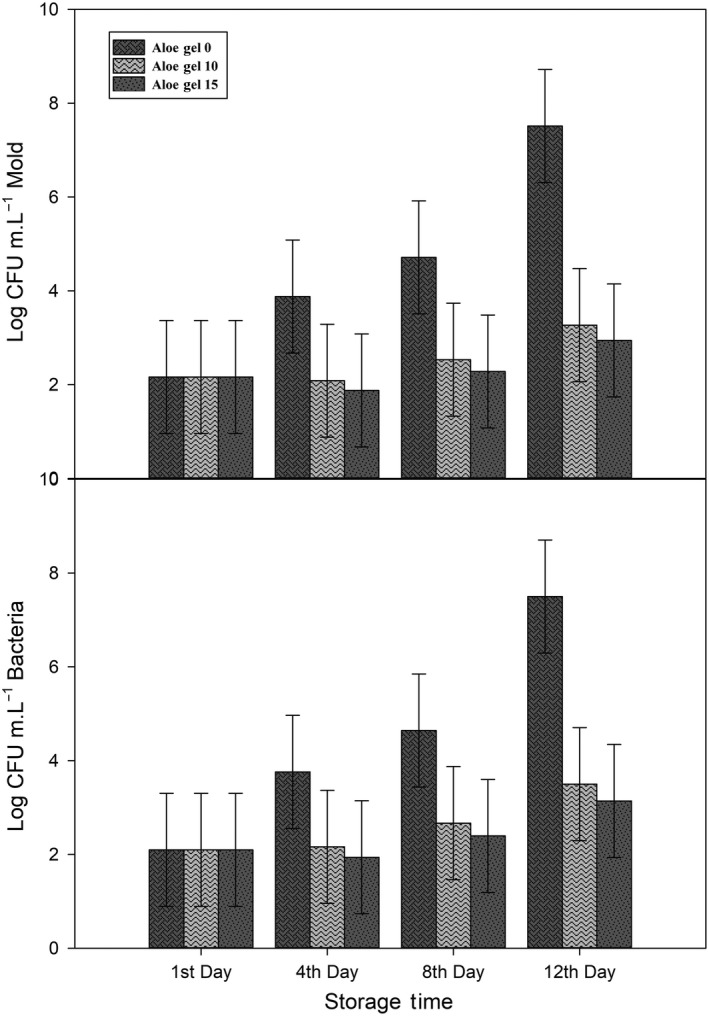
Effect of different Aloe Gel concentration on bacterial and mold contamination (log CFU m.L^−1^) during the storage of pomegranate arils. Each error bar is constructed using a 95% confidence interval of the mean according to Tukey HSD multiple range test

**Figure 7:**
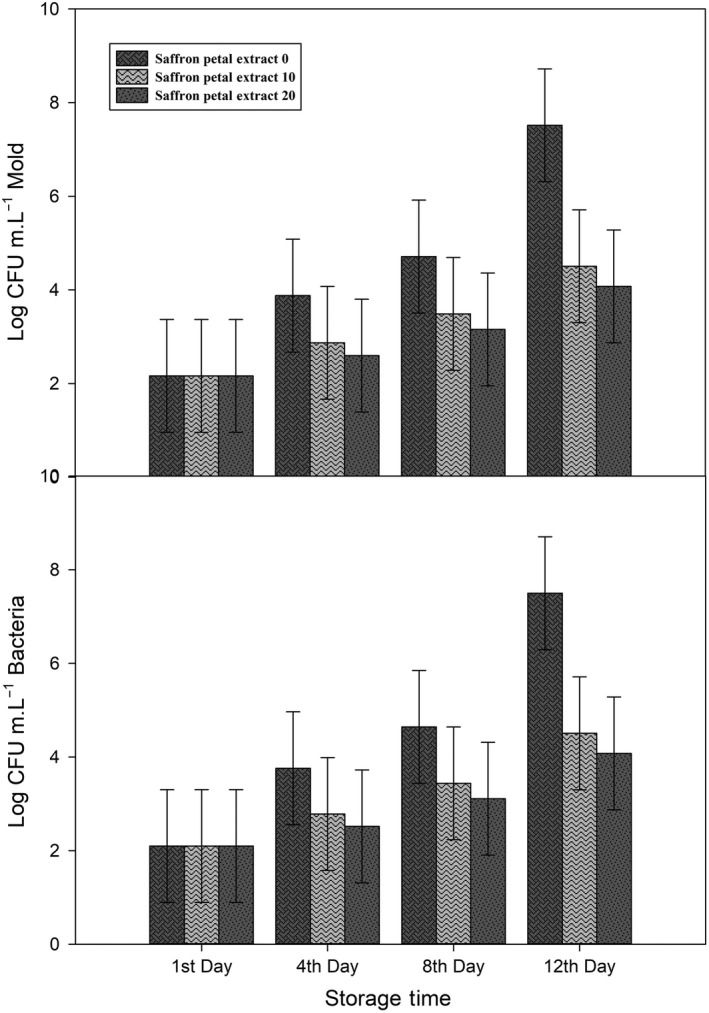
Effect of different saffron petal extract concentrations on bacterial and mold contamination (log CFU m.L^−1^) during storage of pomegranate arils. Each error bar is constructed using a 95% confidence interval of the mean according to Tukey HSD multiple range test

**Figure 8:**
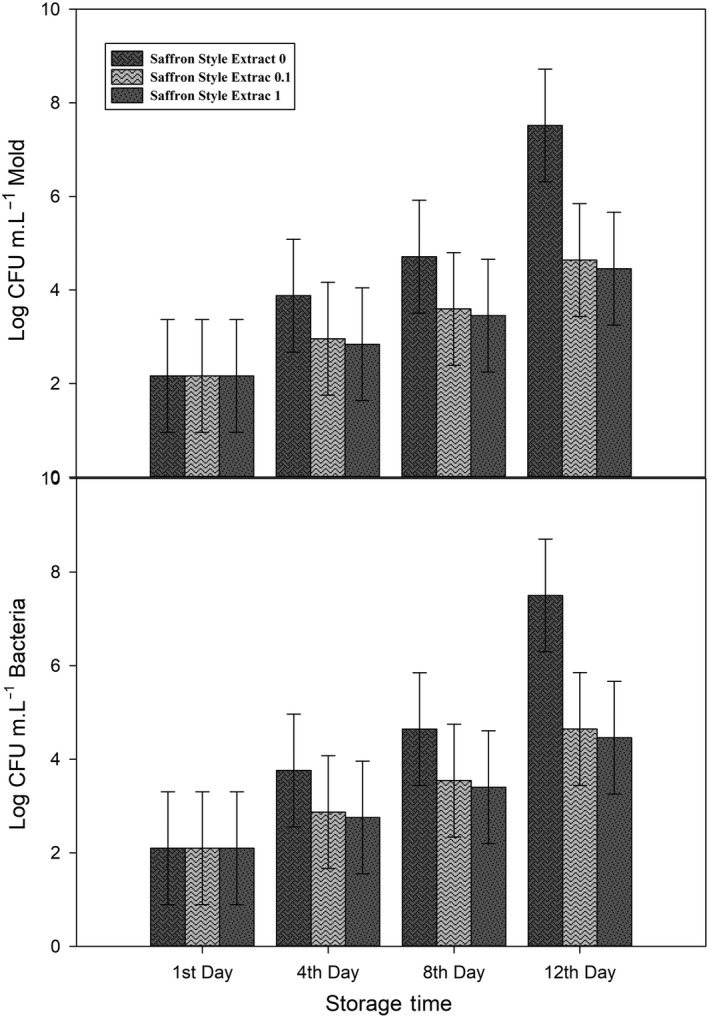
Effect of different saffron style extract concentrations on bacterial and mold contamination (log CFU m.L^−1^) during the storage of pomegranate arils. Each error bar is constructed using a 95% confidence interval of the mean according to Tukey HSD multiple range test

Initial microbial count of bacteria and mold was 2.097 and 2.163 log CFU m.L^−1^, respectively. Through storage time, microbial contamination of arils becomes higher (Figure 6, Figure 7, and Figure 8) and gets to its maximum in the 12th day of storage, 7.49, and 7.52 log CFU m.L^−1^ for bacteria and mold, respectively. All treatments were successful in controlling microbial contamination in both mold and bacteria.

In different studies on pomegranate arils, packaging with different films changed Co_2_ concentration and intercellular pH, which was successfully decreased microbial contamination (Ayhan & Eştürk, 2009; Banda, Caleb, Jacobs, & Opara, 2015; Belay et al., 2018). In this study, Aloe Gel may act like an active‐controlled atmosphere packaging and reduced microbial contamination like them. Similar findings of microbial growth inhibition in pomegranate arils treated with Aloe gel were reported by Martinez‐Romero, 2013, in which the antimicrobial effect of Aloe Gel may reduce microbial contamination on processed arils (Martínez‐Romero et al., 2013).

Both saffron extracts successfully decreased microbial contamination (Figure 7 and Figure 8) and kept them below the acceptable limit (5 log CFU m.L^−1^ for mold and 7 log CFU m.L^−1^ for bacteria) according to Belay et al. (2018). Results of Gandomi *et al.,* 2012 revealed that saffron extracts have antimicrobial effects and could be used as potential sources of natural inhibitors (Gandomi Nasrabadi et al., 2012). In another study, Muzzaffar *et al.* show in vitro inhibitory effects of saffron stigma extracts on both fungi and bacteria and suggest their application in food and pharmaceutical formulations (Muzaffar, Rather, & Khan, 2016). In an unpublished self‐research, we had similar results of lower microbial contamination after application saffron petal and style extracts on *"Jonagold* " apple cubes.

## 4 CONCLUSION

There are so many different treatments used in pomegranate for more extended storage in favorable conditions and minimal defects of fruit quality. While the fresh‐cut industry has pros and cons, in pomegranate, it will provide a possible use of fruit peel in food, health, and cosmetic products. Application of controlled or modified storage successfully increased arils quality in combination with the organic and inorganic compound. Using natural antioxidants and antimicrobials like saffron derivatives (*Safranal*) will lower preservative application in minimal fruit processing and bring more healthy food to the community. Besides their antimicrobial effects, saffron extracts, especially from unused parts of the flower, may also act like nutritional additives for each red or purple fresh‐cut horticultural produce and can increase their nourishment.

## CONFLICT OF INTERESTS

Here, we declare that authors do not have any competing interests.

## AUTHORS' CONTRIBUTIONS

H, K., S, V., contributed substantially to the conception and design of the study, the acquisition of data, the analysis, and interpretation. Both authors have read and approved the manuscript.

## ETHICAL STATEMENTS

This study does not involve any human or animal testings.

## Data Availability

Data of all results will be available through Mendeley Data repository systems: Kaveh, Hamed; vatandoost, safieh (2019), “saffgranate”, Mendeley Data, V1, https://doi.org/10.17632/5wp8zr8kng.1

## ENDNOTE

1 Mass cyanidin‐3‐glucoside equivalent.
